# Decreased expression of interferon-induced protein 2 (IFIT2) by Wnt/β-catenin signaling confers anti-apoptotic properties to colorectal cancer cells

**DOI:** 10.18632/oncotarget.22122

**Published:** 2017-10-26

**Authors:** Tomoyuki Ohsugi, Kiyoshi Yamaguchi, Chi Zhu, Tsuneo Ikenoue, Yoichi Furukawa

**Affiliations:** ^1^ Division of Clinical Genome Research, Advanced Clinical Research Center, Institute of Medical Science, The University of Tokyo, Minato-ku, Tokyo 108-8639, Japan

**Keywords:** IFIT2, colorectal cancer, Wnt, β-catenin, apoptosis

## Abstract

Impaired Wnt signaling pathway plays a crucial role in the development of colorectal cancer through activation of the β-catenin/TCF7L2 complex. Although genes up-regulated by Wnt/β-catenin signaling have been intensively studied, the roles of down-regulated genes are poorly understood. In this study, we explored a global gene expression of colorectal cancer cells transfected with β-catenin siRNAs or a dominant negative form of TCF7L2 (dnTCF7L2), and identified a set of genes down-regulated by Wnt/β-catenin signaling. Among the genes, we focused here on *IFIT2*, a gene encoding interferon-induced protein with tetratricopeptide repeats. A reporter assay using plasmids containing a 5’-flanking region of the gene showed that the reporter activity was enhanced by either transduction of β-catenin siRNA or dnTCF7L2, suggesting that the region is involved in the transcriptional regulation as a downstream of the β-catenin/TCF7L2 complex. Consistent with this result, expression of *IFIT2* was significantly lower in colorectal cancer tissues than that in normal tissues. Exogenous IFIT2 expression decreased cell proliferation and increased apoptosis of colorectal cancer cells. These data suggested that the down-regulation of IFIT2 by Wnt/β-catenin signaling may play a vital role in human colorectal carcinogenesis through the suppression of apoptosis.

## INTRODUCTION

Wnt signaling pathway is associated with maintenance of organogenesis, growth, and homeostasis through the regulation of differentiation, proliferation, and cell fate of epithelial cells [1]. Aberrant Wnt signaling has been linked to many diseases including cancer, fibrosis, and neurodegeneration diseases [2]. One of the key mediators in this pathway is β-catenin. In the absence of Wnt signaling, a multi-molecular complex comprising of β-catenin, APC, Axin, and GSK-3β phosphorylates β-catenin, leading to its degradation in a proteasome-dependent pathway [3]. Upon the binding of Wnt ligands to Frizzled receptors and low density lipoprotein receptor-related protein, Dishevelled proteins are activated and inhibit the activity of GSK-3β [4]. Consequently, degradation of β-catenin is suppressed, and the accumulated β-catenin is translocated into the nucleus, where it binds to transcription factors such as T-cell factor/lymphoid enhancer factor (TCF/LEF). The TCF/LEF family is then activated and transactivates the expression of their target genes. In 93% of colorectal tumors, Wnt/β-catenin signaling is deregulated mainly due to the inactivation of *APC* or activating mutations of *CTNNB1* [5], which results in the elevated expression of target genes of the β-catenin/TCF complex.

Many efforts have been made to identify direct downstream targets of β-catenin/TCF such as c-myc [6], cyclin D1 [7], MMP-7 (matrilysin) [8], LGR5 [9], urokinase-type plasminogen activator receptor (uPAR) [10], connexin 43 [11], CD44 [12], AF17 [13], ENC1 [14], Laminin-5γ2 [15], PPAR-delta [16], Claudin-1 [17], and MT1-MMP [18]. To date, regulatory mechanisms and molecular functions of these target genes have been well studied in cancer cells. For example, c-myc [19] and cyclin D1 [20] regulate cell proliferation and cell cycles progression, respectively. LGR5 is involved in the maintenance of stemness [21]. Functional analyses of these target genes have contributed to understanding the role of β-catenin-dependent Wnt signaling in carcinogenesis. However, the roles of down-regulated downstream genes by the signaling remain largely unknown.

The interferon-induced proteins with tetratricopeptide repeats (IFITs) consist of four members, IFIT1, IFIT2, IFIT3, and IFIT5, in humans. Induced by interferons, or through the membrane-bound Toll-like receptors (TLRs), or the cytoplasmic RIG-1-like receptors (RLRs), IFITs play a crucial role in host antiviral defense as an innate immune response. IFIT1 and IFIT2 bind to the multi-subunit eukaryotic translation initiation factor 3 (eIF3) [22], interfere with the assembly of the preinitiation complex containing the 40S ribosomal subunit, eIF3, eIF2/GTP/Met-tRNA, and eIF4F [23], and inhibit translation initiation. In addition, IFIT1, IFIT2 and IFIT3 form a complex that recognizes viral RNA lacking 2'-O methylation on their cap structures or displaying a 5′-triphosphate group (PPP-RNA), a molecular signature that distinguishes viral RNA from host RNA [24]. Interestingly, IFIT1 and IFIT2 associate with MITA (mediator of IRF3 activation), and induce apoptosis via the mitochondrial pathway [25, 26]. Moreover, IFIT2 elicits apoptotic cell death independent of IFN stimulation [26]. On the other hand, IFIT3 interacts with IFIT2, and negatively regulates the apoptotic effects of IFIT2 [22]. Although roles of IFITs in viral infection have been intensively investigated, studies regarding their roles in carcinogenesis have been limited.

In this study, we identified IFIT1 and IFIT2 as genes down-regulated by Wnt signaling in colorectal cancer (CRC) cells, and clarified that inhibition of IFIT2 may play a role in the proliferation and anti-apoptotic properties of CRC cells. Our findings may be helpful for the profound understanding of colorectal carcinogenesis.

## RESULTS

### Genes down-regulated by the β-catenin/TCF7L2 complex in CRC

**Figure 1:**
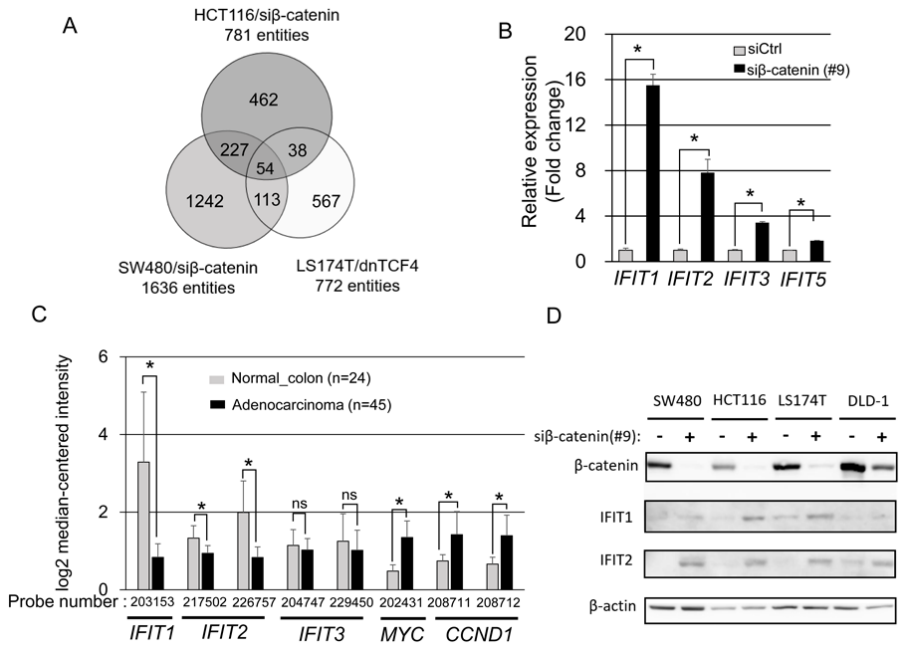
Identification of *IFIT1* and *IFIT2* as downstream genes repressed by Wnt/β-catenin **(A)** Integration of three microarray data identified 53 commonly upregulated genes and one non-coding RNA by inhibition of the β-catenin/TCF7L2 complex. **(B)** Expression levels of *IFITs* in SW480 cells treated with β-catenin or control siRNA for 48 h were determined by real time-PCR. Expression of *GAPDH* was used as an internal control. The data represents mean ± SD from three experiments. Asterisk indicates *p*<0.05. **(C)** Expression of *IFIT1, IFIT2, IFIT3, MYC, and CCND1* in CRC tissues obtained from a public database, Gene Expression Atlas (GSE20916). **(D)** Increased IFIT1 and IFIT2 proteins by the knockdown of β-catenin in CRC cell lines. The lysates from CRC cells transfected with β-catenin or control siRNA for 48 h were subjected to western blotting. Expression of β-actin served as an internal control.

To identify genes down-regulated by the β-catenin/TCF7L2 complex, we searched for those up-regulated by the suppression of β-catenin or TCF7L2. First, we performed gene expression profile analyses of SW480 and HCT116 cells treated with β-catenin siRNA or control siRNA. These analyses identified 781 and 1636 entities whose expression levels were elevated greater than two-fold by the β-catenin siRNA compared with control siRNA in HCT116 and SW480 cells, respectively. In addition, we identified 772 entities whose expression was enhanced more than two-fold by a dominant-negative form of TCF7L2 (dnTCF7L2) in LS174T cells using public expression profile data (GSE46465) [27]. Taking these data together, we identified a total of 54 entities that were commonly up-regulated in response to the suppression of the β-catenin/TCF7L2 complex (Figure 1A and Supplementary Table 1). The 54 entities include 53 genes and one non-coding RNA, and the top 20 genes induced by the suppression of the complex are shown in Table 1.

**Table 1:** List of the top 20 up-regulated genes in CRC cells treated with β-catenin or dnTCF7L2

Gene symbol		Fold change			
		SW480 (siβ-catenin)	HCT116 (siβ-catenin)	LS174T (dnTCF7L2)	Average
1	*ANGPTL4*	40.67	4.71	10.60	18.66
2	*ANXA8*	32.78	2.31	5.38	13.49
	*ANXA8L1*				
	*ANXA8L2*				
3	*ANXA8L2*	32.78	2.31	5.25	13.45
	*ANXA8L1*				
4	*KPNA7*	23.81	13.93	2.52	13.42
5	*ANXA8L1*	32.78	2.31	4.53	13.21
	*ANXA8L2*				
6	*IFIT1*	20.39	3.13	5.26	9.59
7	*C10orf10*	18.29	4.39	2.73	8.47
8	*MB*	2.08	12.68	10.24	8.34
9	*CD68*	2.79	4.88	16.45	8.04
10	*PLAU*	8.62	3.22	11.18	7.67
11	*KLHL30*	7.34	9.08	4.31	6.91
12	*IFIT2*	11.36	3.89	5.31	6.86
13	*PTAFR*	7.03	2.07	11.37	6.82
14	*HMOX1*	3.30	4.79	9.99	6.02
15	*BMF*	4.20	2.65	11.03	5.96
16	*FGFBP1*	9.59	4.67	3.24	5.83
17	*AHNAK2*	5.52	3.26	7.93	5.57
18	*ELF3*	7.73	3.95	4.38	5.36
19	*APOL6*	3.00	10.06	2.18	5.08
20	*MVP*	9.27	2.64	2.22	4.71

Genes were sorted based on average fold changes across the three conditions.

### Biological significance of a set of genes down-regulated by the complex

To understand the biological significance of genes down-regulated by the activation of Wnt-signaling in the cells, Gene Set Enrichment Analysis were carried out with curated gene sets in the Molecular Signatures Database (http://software.broadinstitute.org/gsea/msigdb/index.jsp), and the 54 down-regulated entities were analyzed (Table 2). The genes were found to be significantly associated with interferon signaling, cytokine signaling in the immune system, and interferon-α/β signaling, suggesting that Wnt signaling might affect interferon-mediated immune responses.

**Table 2:** Gene Set Enrichment Analysis of up-regulated genes by suppression of the β-catenin/TCF complex

Gene Set	Description	Genes in Overlap	Gene symbol	p value	FDR
REACTOME_INTERFERON_SIGNALING	Interferon signaling	4	*IFIT1, IFIT2, IFIT3, PTAFR*	2.38 e-5	2.83 e-2
REACTOME_INTERFERON_ALPHA_BETA_SIGNALIALING	Interferon α/β signaling	3	*IFIT1, IFIT2, IFIT3*	4.26e-5	2.83e-2
NABA_MATRISOME	Extracellular matrix and extracellular matrix -associated proteins	7	*FGF1, PLAU, ANGPTL4*, *FGFBP1, SEMA7A*, *SEMA4B, LAMB3*	9.09e-4	4.03e-2
NABA_MATRISOME_ASSOCIATED	ECM-associated proteins including ECM-affilaited proteins, ECM regulators and secreted factors	6	*FGF1, PLAU, ANGPTL4 FGFBP1, SEMA7A*, *SEMA4B*	1.3 e-4	4.31e-2
REACTOME_CYTOKINE_SIGNALING_IN_IMMUNE_NE_SYSTEM	Cytokine signaling in immune system	4	*IFIT1, IFIT2, IFIT3, PTAFR*	1.85 e-4	4.92e-2

### Expression of IFITs in CRC

We found that two interferon-induced genes, *IFIT1* and *IFIT2*, were included in the top 20 in the list of down-regulated genes (Table 1). In addition*, IFIT3* was in the top 30 genes. Subsequent quantitative RT-PCR analysis determined that knockdown of β-catenin by siRNA increased *IFIT1* (15.5-fold), *IFIT2* (7.8-fold), and *IFIT3* (3.4-fold) expression, compared with control siRNA in SW480 cells (Figure 1B).

Since activation of the β-catenin/TCF7L2 complex is a frequent feature observed in CRC, we compared expression of *IFIT1, IFIT2*, and *IFIT3*, and that of *MYC* and *CCND1*, two direct target genes of the complex, between colorectal tumor tissues and normal colonic mucosae using the Oncomine cancer profiling database (GSE20916 in https://www.oncomine.org). As shown in Figure 1C, expression levels of *MYC* and *CCND1* were significantly elevated in tumor tissues than those in normal tissues (2.80-fold with *MYC* probe #202431, *p*=4.0x10^-14^; 1.91-fold with *CCND1* probe #208711, *p*=8.96x10^-7^; and 2.11-fold with *CCND1* probe #208712, *p*=8.79x10^-9^). On the other hand, expression levels of *IFIT1* and *IFIT2* were significantly down-regulated in colorectal adenocarcinoma compared with normal tissues (-3.95-fold with *IFIT1* probe #203153, *p*=6.3x10^-13^; -1.42-fold with *IFIT2* probe #217502, *p*=2.91x10^-8^, and -2.40-fold with *IFIT2* probe #226757, *p*=1.1x10^-12^) [28]. In addition, two datasets (GSE4183 [29] and GSE8671 [30]) revealed that expression levels of *IFIT1* and *IFIT2* were also down-regulated in colorectal adenoma tissues compared with normal tissues (Supplementary Figure 1). However, the expression of *IFIT3* was not significantly altered (-1.11-fold, *p*=0.17 with *IFIT3* probe #204747: and -1.23-fold with *IFIT3* probe #229450, *p*=0.12) in colorectal adenocarcinoma (Figure 1C), suggesting that other factor(s) may be involved in the regulation of *IFIT3* expression in colorectal tumors. Western blotting corroborated that knockdown of β-catenin increased IFIT1 and IFIT2 proteins in SW480, HCT116, LS174T, and DLD-1 cells. (Figure 1D). These data indicated that IFIT1 and IFIT2 proteins are down-regulated in colorectal cancer cells by Wnt signaling.

To clarify the negative regulation of *IFIT1* and *IFIT2* by the Wnt signaling, we introduced an activating form of β-catenin lacking exon3 (pcDNA-CTNN1BΔexon3) [31] into HeLa cells that have normal Wnt signaling pathway, and analyzed *AXIN2* expression that is another target of the β-catenin/TCF7L2 complex. Quantitative RT-PCR analysis determined that overexpression of β-catenin increased *AXIN2* expression by 16-fold and that it reduced expression of *IFIT1* and *IFIT2* (0.3-fold and 0.4-fold, respectively) (Supplementary Figure 2). These data strengthened that the expression of IFIT1 and IFIT2 is negatively regulated by Wnt signaling.

### Regulation of *IFIT1* and *IFIT2* promoter activities

**Figure 2:**
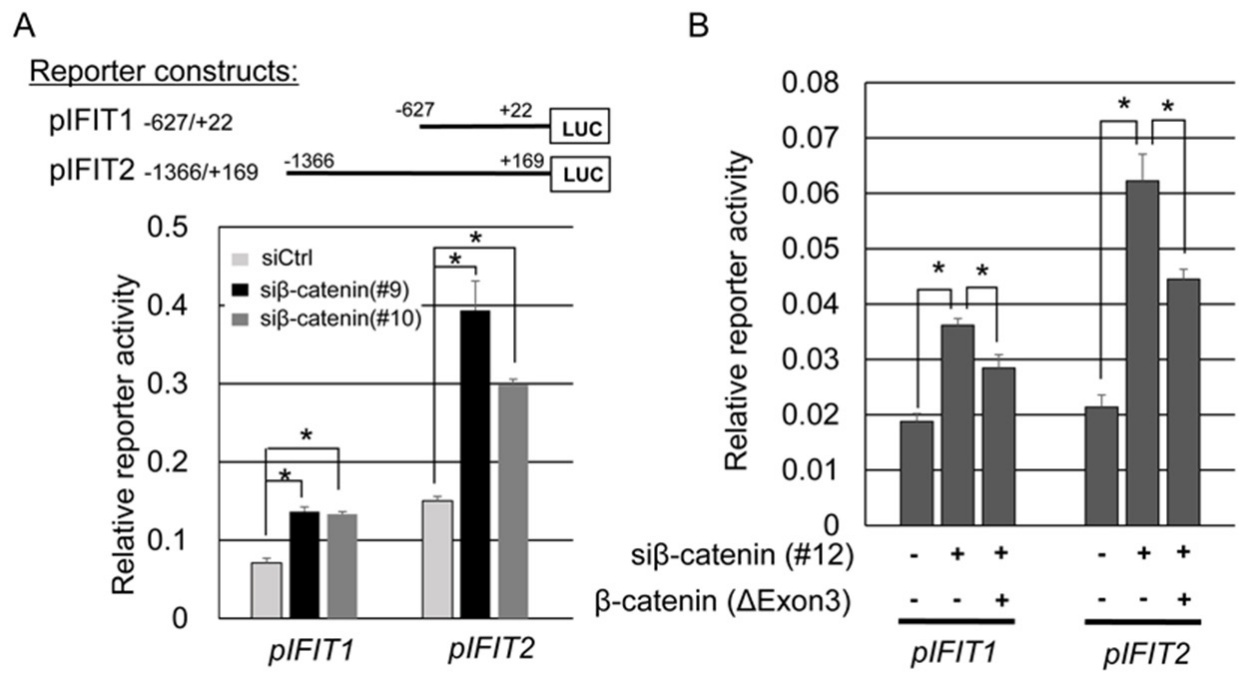
Association between the reporter activities of IFIT1 and IFIT2, and β-catenin expression **(A)** Reporter activities of pIFIT1 and pIFIT2 in the presence or absence of β-catenin siRNA in SW480 cells. The cells were transfected with the indicated reporter plasmids in combination with different β-catenin siRNAs (#9 or #10) or control siRNA. The data represents mean ± SD from three experiments. An asterisk indicates *p*<0.05. **(B)** Suppression of the induced reporter activities of pIFIT1 and pIFIT2 by β-catenin siRNA (#12) with an activating mutant form of β-catenin in SW480 cells. The mutant form of β-catenin lacks the sequence targeted by β-catenin siRNA (#12), and thus is not suppressed by the siRNA. The luciferase activities represent mean ± SD from three-independent cultures. An asterisk indicates *p*<0.05.

To clarify transcriptional regulation of *IFIT1* and *IFIT2* by the β-catenin/TCF7L2 complex, we carried out a reporter assay using plasmids containing their 5’-flanking regions (pIFIT1 -627/+22 and pIFIT2 -1366/+169) in SW480 cells. Dual-luciferase assay revealed that the reporter activity of pIFIT1 was augmented 1.92- and 1.88-fold by β-catenin siRNA#9 and siRNA#10, respectively, and that of pIFIT2 was augmented 2.62- and 1.98-fold by siRNA#9 and siRNA#10, respectively (Figure 2A). To examine whether β-catenin can repress the enhanced expression and reporter activities of IFIT1 and IFIT2, we transfected the cells with plasmids that express the activating mutant form of β-catenin lacking exon3 together with β-catenin siRNA#12 that targets a sequence in exon3. As shown in Figure 2B, the enhanced reporter activities of IFIT1 and IFIT2 by β-catenin siRNA#12 were suppressed in part by the expression of β-catenin, supporting that β-catenin play a suppressive role in the transcriptional activities of *IFIT1* and *IFIT2* in CRC cells. Since β-catenin has been known to function as a coactivator of TCF transcription factors, we assume that *IFIT1* and *IFIT2* are down regulated by β-catenin/TCF complex through unknown mechanism(s). Two prediction programs, JASPAR (http://jaspar.genereg.net/) and TFBIND (http://tfbind.hgc.jp/), did not predict TCF/LEF binding motif(s) in their promoter regions (data not shown).

### Involvement of IFIT2 in apoptosis

**Figure 3:**
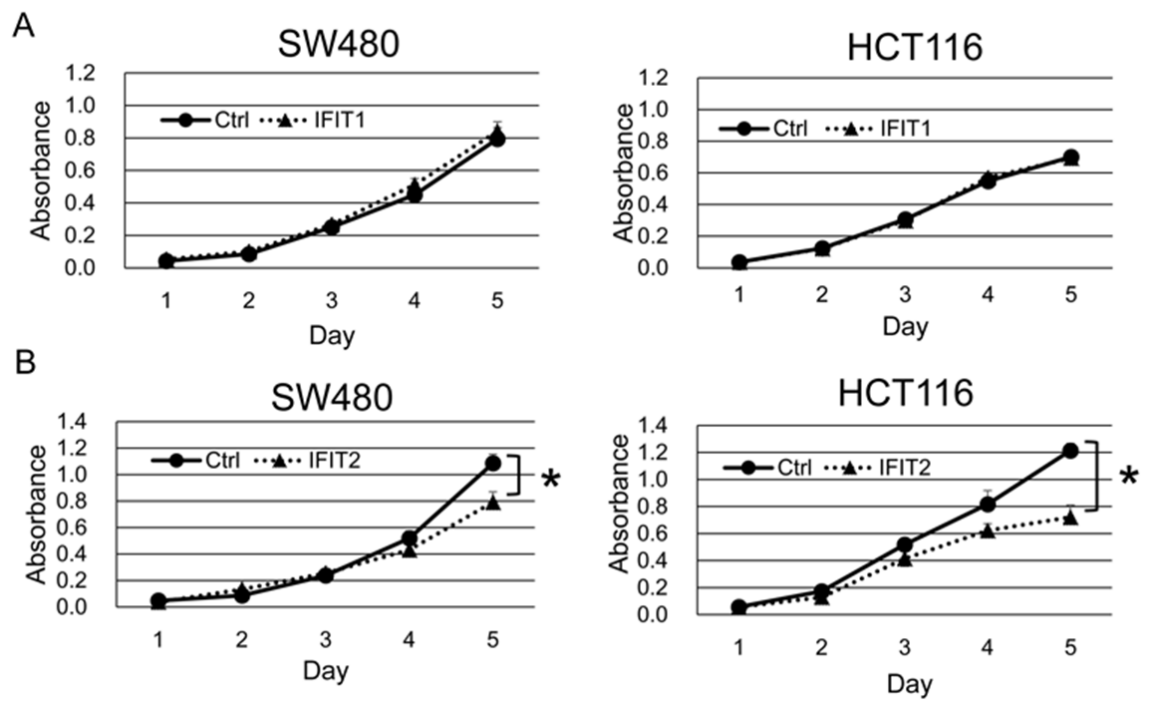
Effect of IFIT1 and IFIT2 on proliferation of CRC cells The proliferation of SW480 and HCT116 cells stably expressing IFIT1 **(A)** or IFIT2 **(B)** was measured by WST-8 assay. A significant difference was determined by two-way ANOVA.

**Figure 4:**
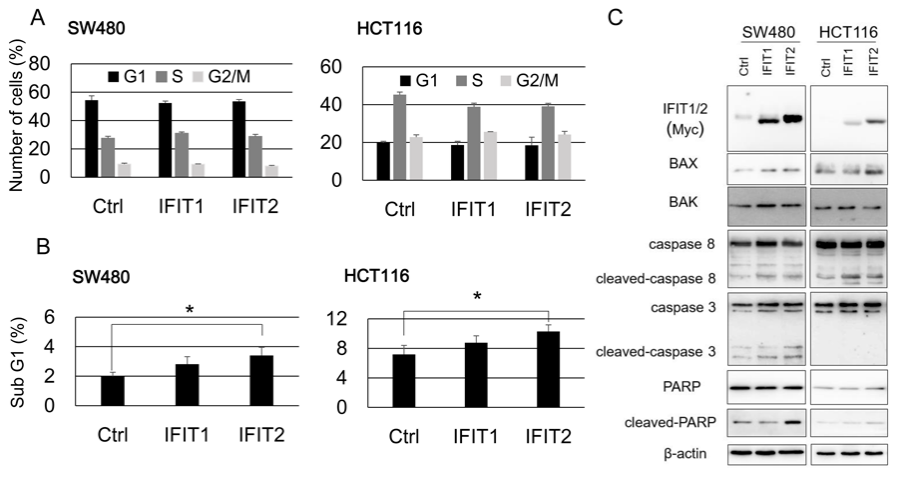
Effect of IFIT1 and IFIT2 on the apoptosis of CRC cells **(A)** Cell cycle analysis of the cells expressing IFIT1 or IFIT2 was performed by FACS. **(B)** Comparison of sub-G1 population in the cells expressing IFIT1 or IFIT2 with control cells. An asterisk indicates *p*<0.05. **(C)** BAX, BAK, and cleavage of caspase-8, caspase-3, and PARP, were analyzed by western blot analysis. Expression of β-actin served as an internal control.

To explore the function of IFIT1 and IFIT2 in CRC cells, we established SW480 and HCT116 cells that stably express exogenous IFIT1 and IFIT2 using the retrovirus transduction system. Exogenous expression of IFIT2, but not IFIT1, significantly suppressed the proliferation of SW480 and HCT116 cells (Figure 3A and 3B). Subsequent cell cycle analysis revealed that their overexpression did not alter the population of G1, S, or G2/M phases. However, the sub-G1 population was significantly increased by IFIT2 in SW480 and HCT116 cells (Figure 4A and 4B), suggesting that induction of apoptosis by IFIT2 may be associated with the suppressed growth of CRC cells. In addition, we found that overexpression of IFIT2 increased the expression of BAX and cleaved forms of caspase-8 and PARP in SW480 and HCT116 cells, suggesting that IFIT2 may increase the sensitivity of cells to apoptosis through the mitochondria pathway. However, cleaved form of caspase-3 was not detected in HCT116 cells but detected in SW480 cells. Response of procaspase-3 to IFIT2 might be different between SW480 and HCT116 cells (Figure 4C). On the other hand, IFIT2 did not enhance the apoptosis induced by camptothecin (an inhibitor of topoisomerase I) or γ-irradiation in SW480 and HCT116 cells (Supplementary Figure 3). These data imply that IFIT2 might not affect the cell viability to DNA-damaging insults.

## DISCUSSION

In this study, we discovered that IFIT1 and IFIT2 are negatively regulated by Wnt signaling in CRC cells and that suppressed expression of IFIT2 may confer pro-survival properties to cancer cells.

IFIT2 or ISG54 (Interferon stimulated gene 54) is a protein of approximately 54 kDa, which forms a complex with IFIT1 and IFIT3. Stawowczyk and colleagues reported that exogenous expression of IFIT2 suppressed proliferation of HeLa cells and promoted their cell death through apoptosis [26]. They also found that Bak and/or Bax were indispensable for the IFIT2-inducted apoptosis, and that Bcl-xl prevented the induction of apoptosis. These results suggested that IFIT2 stimulated apoptosis through the regulation of mitochondrial membrane permeability. In agreement with their view, overexpression of IFIT2 increased the expression of BAX, and cleaved forms of PPAR and caspase-8 in CRC cells, suggesting that IFIT2 plays a role in the apoptosis via mitochondria-mediated pathway. Therefore, reduced expression of IFIT2 might render anti-apoptotic properties to colorectal cancer cells.

Of note, they additionally showed that IFIT3 but not IFIT1 counteracted the induction of apoptosis by IFIT2. Since activation of Wnt signaling in cancer cells decreased both IFIT1 and IFIT2, co-repression of these genes may confer the pro-survival properties to the cancer cells by increasing resistance to apoptotic signals. We found that the effect of aberrant Wnt signaling on the repression of IFIT3 is mild compared to that of IFIT1 and IFIT2, and that IFIT3 expression was not decreased in CRC tissues. These data may imply that IFIT1 and IFIT2 are the main targets of Wnt signaling among the human IFITs in CRC.

In addition, Feng *et al.* reported that IFIT2 is post-transcriptionally regulated by miR-645, a microRNA that is abundantly expressed in adenocarcinoma of gastric esophageal junction (AGEJ) [32]. They proved that the expression levels of IFIT2 were negatively correlated with those of miR-645 in clinical AGEJ tissues, and showed that IFIT2 expression was significantly lower in tumors greater than five centimeters in size compared with those less than five. Importantly, they found that exogenous expression of IFIT2 significantly suppressed the growth of gastric cancer cells, and promoted apoptosis in the cells treated with Adriamycin. Recently, another group reported that LINC00161, a long non-coding RNA, sensitized cisplatin-induced apoptosis through the modulation of the miR-645-IFIT2 pathway in osteosarcoma [33]. These data suggest that decreased expression of IFIT2 may confer growth advantage and anti-apoptotic characteristics to cancer cells, which is in good agreement with our findings in this study.

Importantly, this is the first report showing that IFIT1 and IFIT2 are negatively regulated by Wnt signaling in colon cancer. Expression of IFITs are induced by viral and bacterial infection [34], type I IFN including IFN-α/β [35], and a variety of cellular stresses such as DNA damage [36]. Although IFIT2 expression is regulated by miR-645 in a post-transcriptional manner as described previously, signal transduction pathways including IFNβ-dependent pathways, IFN-regulatory factor 3 (IRF3)-dependent pathways, NF-κB pathway, and JAK-STAT pathway are involved in the regulation of IFITs expression [22]. In addition, IFIT2 is also induced by the recruitment of IRF transcription factors such as IRF1, IRF3, IRF5, IRF7, and IRF9 [37–40]. Future studies may disclose a novel link between Wnt signaling and IFN-regulatory factors.

Although IFIT1 and IFIT2 may not be direct targets of the β-catenin/TCF transcriptional complex, suppressed expression of these molecules may be involved in carcinogenesis through the activation of anti-apoptotic properties. Further studies of their regulation may contribute to a better understanding of colorectal carcinogenesis and the development of strategies to enhance apoptotic effect of anti-cancer drugs.

## MATERIALS AND METHODS

### Cell culture

Human CRC cell lines, HCT116, SW480, DLD-1, LS174T and human cervix cell lines, HeLa were purchased from the American Type Culture Collection (Manassas, VA). All cells were grown in appropriate media (McCoy’s 5a Medium Modified for HCT116; RPMI-1640 for DLD-1; Leibovitz’s L-15 for SW480; EMEM for HepG2, HeLa, and LS174T) supplemented with 10% FBS (ThermoFisher, Waltham, MA), and antibiotic/antimycotic solution (Sigma, St. Louis, MO).

### Gene silencing

Three β-catenin siRNAs (ON-TARGETplus SMARTpool siRNA L-003482-00, *#*9, *#*10, and *#*12) and control siRNA (ON-TARGETplus Non-targeting Pool *#*D-001810-10) were purchased from GE Dharmacon (Lafayette, CO). Target sequences of the siRNAs are shown in Supplementary Table 2. HCT116 or SW480 were seeded a day before the treatment with siRNA, and transfected with 15 nM of *β*-catenin or control siRNA using Lipofectamine RNAiMAX (Thermo Fisher Scientific). Forty-eight hours after the transfection, RNA and proteins were extracted from the cells. The silencing effect of β-catenin siRNAs was evaluated by quantitative RT-PCR and western blotting.

### Gene expression analysis

Total RNA was extracted from the cells treated with *β*-catenin or control siRNA using RNeasy Plus mini Kit (Qiagen, Valensia, CA), and RNA integrity was assessed by 2100 Bioanalyzer (Agilent Technologies, Santa Clara, CA). Two-hundred microgram of total RNA were amplified and labeled using a Low Input QuickAmp Labeling Kit according to the manufacturer’s protocols (Agilent Technologies). Expression profiles were analyzed using SurePrint G3 Human Gene Expression 8x60K Microarray according to the supplier’s recommendations (Agilent Technologies). Subsequent data processing was performed using the GeneSpring GX13.1 (Agilent Technologies). Additionally, gene set enrichment analysis was performed using the Molecular signatures database (MSigDB, http://www.broadinstitute.org/gsea/msigdb/index.jsp) with gene sets derived from Canonical pathways, BioCarta, KEGG, and Reactome.

### Quantitative PCR

Complementary DNA (cDNA) was synthesized from one μg of total RNA with Transcriptor First Strand cDNA Synthesis Kit (Roche Diagnostics GmbH, Mannheim, Germany). Real-time PCR was performed using qPCR Kapa SYBR Fast ABI Prism Kit (Kapa Biosystems, Wilmington, MA) with sets of primers for *IFIT1, IFIT2*, and *IFIT3* on StepOnePlus (Thermo Fisher Scientific). Sequences of the primers used are shown in Supplementary Table 3. The levels of transcripts were determined by the relative standard curve method, and *GAPDH* was used as internal control.

### Western blotting

Total protein was extracted from cultured cells using radioimmunoprecipitation assay (RIPA) buffer (50 mM Tris-HCl, pH8.0, 150 mM sodium chloride, 0.5% sodium deoxycholate, 0.1% sodium dodecyl sulfate, 1.0% NP-40) supplemented with a Protease Inhibitor Cocktail Set III (Calbiochem, San Diego, CA). Protein concentration was determined by BCA Protein Assay Kit (Thermo Scientific, Rockford, IL). Protein (30-50 μg/lane) was separated by 10% SDS-PAGE and transferred on to a nitrocellulose membrane (GE Healthcare, Buckinghamshire, UK). After the blocking with 5% milk powder in TBS-T ( Tris-buffered saline - Tween20) for 1 hour, the membranes were incubated over night with primary antibodies including anti-β-catenin (9582, Cell Signaling Technology, Danvers, MA), anti-β-actin (A5441, Sigma), anti-IFIT1 (HPA055380, sigma), anti-IFIT2 (12604-1-AP, Proteintech Group, Chicago, MA), anti-PARP (9532, Cell Signaling Technology), anti-cleaved PARP (9541, Cell Signaling Technology), anti-Bax (sc-493, Santa Cruz Biotechnology, Dallas, TX), anti-Bak (sc-832, Santa Cruz Biotechnology), anti-caspase-3 (9662, Cell Signaling Technology), and anti-caspase-8 antibodies (M032-3, MBL, Nagoya, Japan). Horseradish peroxidase-conjugated goat anti-mouse or anti-rabbit IgG (GE Healthcare) served as the secondary antibody for the ECL Detection System (GE Healthcare).

### Reporter assay

The 5′-flanking regions of *IFIT1* (-627/+22) and *IFIT2* (-1366/+169) were amplified by PCR, and the DNA fragments were cloned into pGL3-Basic vector (Promega, Madison, WI) to generate reporter plasmids (pIFIT1 -627/+22 and pIFIT2 -1366/+169, respectively). The primer sequences are shown in Supplementary Table 4. Cells were transfected with the reporter plasmids and pRL-TK (Promega) in combination with control or *β*-catenin siRNA. Reporter assay was carried out using a dual-luciferase reporter assay system (Toyo Ink, Tokyo, Japan) according to the supplier’s recommendations.

### Retroviral transduction

The pMXs retroviral vectors carrying the *IFIT1* and *IFIT2* genes were constructed by the insertion of DNA fragments encoding full-length *IFIT1* (NM_001548) and *IFIT2* (NM_001547), respectively. The primer sequences used for the amplification are shown in Supplementary Table 4. Retroviral particles were produced by the transfection of PLAT-A packaging cells with pMX-IFIT1, pMX-IFIT2, or pMX-EGFP (a kind gift from Dr. Kitamura, The University of Tokyo). Culture supernatants containing the retrovirus were collected and used to transduce the RNA in the CRC cells. The cells expressing each gene were selected in medium containing puromycin (HCT116: 1 μg/ml and SW480: 2.5 μg/ml).

### Cell cycle analysis

SW480 and HCT116 cells stably expressing IFIT1, IFIT2, or control EGFP were fixed with 70% ethanol at -20°C. The cells were treated with RNase A (2 mg/ml) at 37°C for 30 min, and incubated with propidium iodide (20 μg/ml) in PBS at room temperature for 30 min. After filtration with nylon mesh, the cells were applied for flow cytometric analysis (FACSCalibur, BD Biosciences, Franklin Lakes, NJ).

### Cell proliferation assay

Cell proliferation assay was carried out by water soluble tetrazolium salts (WST)-based colorimetric method using Cell-counting kit-8 according to the supplier’s protocol (Dojindo, Kumamoto, Japan). Absorbance was measured at 450 nm using FLUOstar OPTIMA (BMG Labtechnologies, GmbH, Germany).

### Statistical analysis

Statistical analysis was performed by Student's t-test with Benjamini-Hochberg correction for the analysis of gene expression profiles. The unpaired Student’s t-test and Dunnett’s test were used for the statistical analysis of RT-PCR and reporter assay data. The data of cell proliferation was analyzed by two-way ANOVA.

## SUPPLEMENTARY MATERIALS FIGURES AND TABLES




