# Supplementary material for: Decreased expression of interferon-induced protein 2 (IFIT2) by Wnt/β-catenin signaling confers anti-apoptotic properties to colorectal cancer cells
Source: Oncotarget. 2017 Oct 26;8(59):100176–86. doi: 10.18632/oncotarget.22122 (PMC5725011; doi:10.18632/oncotarget.22122)
Supplement: Supplementary file 1 [file oncotarget-08-100176-s001.pdf]

Decreased expression of interferon-induced protein 2 (IFIT2) by Wnt/β-catenin signaling confers anti-apoptotic properties to colorectal cancer cells

SUPPLEMENTARY MATERIALS

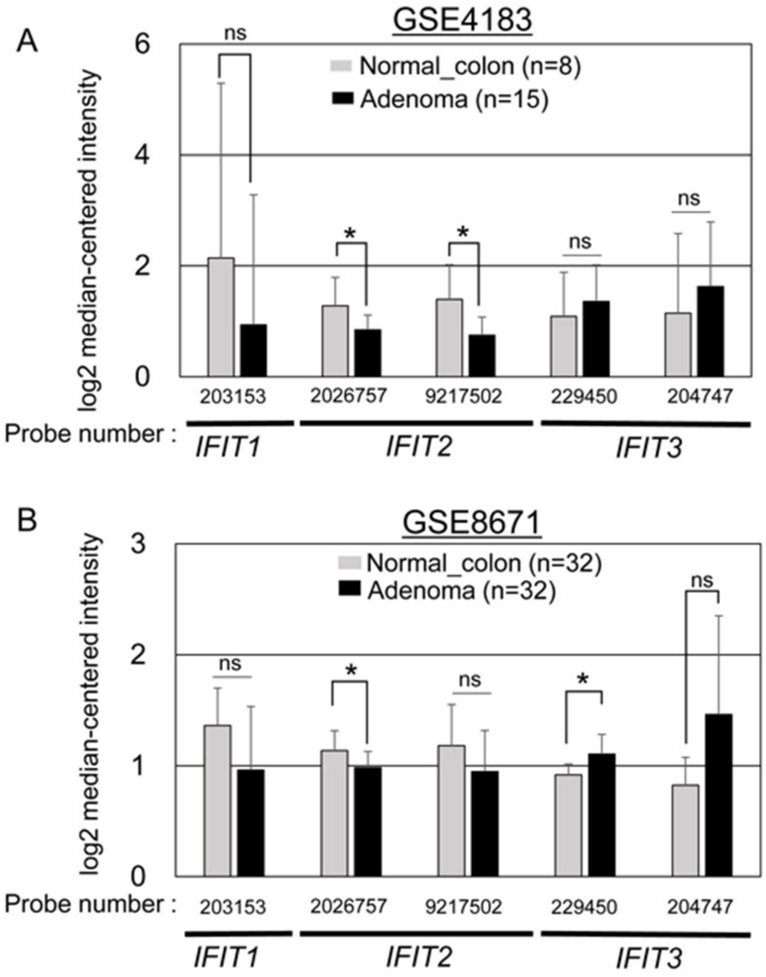

Supplementary Figure 1: Expression of *IFIT1, IFIT2, and IFIT3* in colorectal adenomas obtained from a public database. Expression profiling by microarray is analyzed with GEO2R to compare normal colon and colon adenoma. **(A)** GSE4183 (normal colon: n=8, colon adenoma: n=15), **(B)** GSE8671 (normal colon: n=32, colon adenoma: n=32).

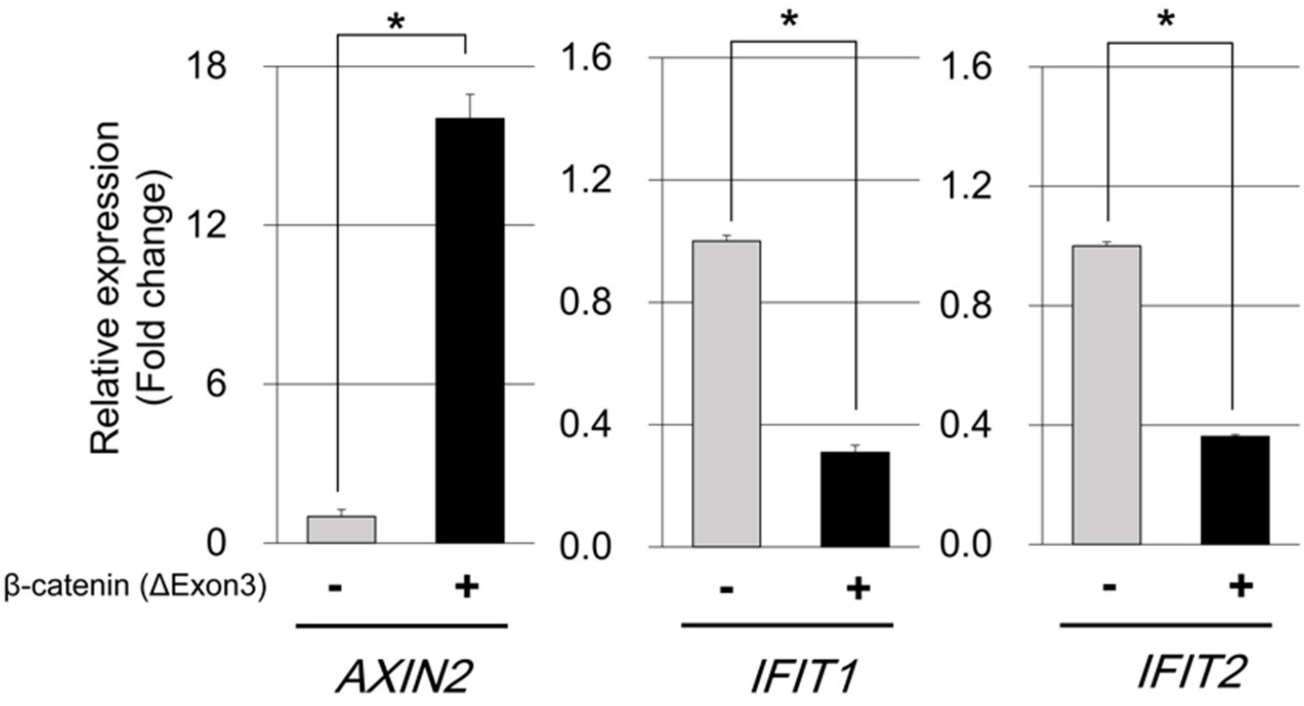

Supplementary Figure 2: Over-expression of β-catenin decrease *IFIT1* and *IFIT2* mRNA expression in HeLa cells. The data represents mean ± SD from three experiments. Asterisk indicates $p<0.05$.

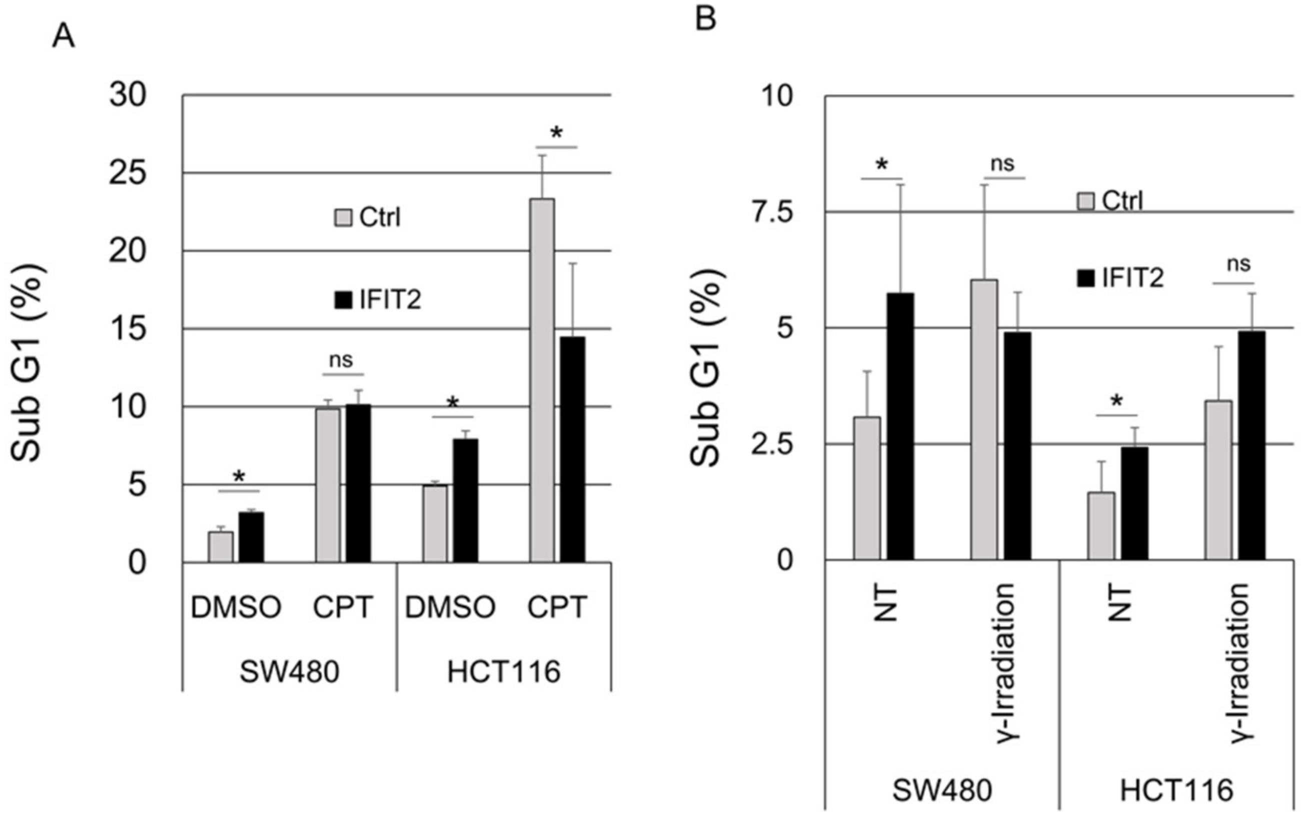

Supplementary Figure 3: IFIT2 did not alter sensitivity to apoptotic stimuli. Comparison of sub-G1 population between the cells expressing IFIT2 and the control cells. **(A)** The cells treated with camptothecin (CPT, 0.5μM). An asterisk indicates $p<0.05$. **(B)** The cells exposed to γ-irradiation (5 Gy). An asterisk indicates $p<0.05$.

Supplementary Table 1: List of up-regulated genes by the treatment with dnTCF4 or β-catenin siRNA

See Supplementary File 1

Supplementary Table 2: mRNA sequences targeted by β-catenin siRNAs

siRNA	Sequence
Control	ON-TARGETplus Non-targeting Control Pool #D-001810-10
β-catenin pool	ON-TARGETplus SMARTpool siRNA, L-003482-00
β-catenin #9	GAUCCUAGCUAUCGUUCUU
β-catenin #10	UAAUGAGGACCUAUACUUA
β-catenin #12	GGUACGAGCUGCUAUGUUC

Supplementary Table 3: Primers for real-time PCR

Gene symbol	Strand	Sequence (5' to 3')
IFIT 1	F	ggaccctgaaaaccctgaat
	R	tgtggctaatttaaagccatcc
IFIT 2	F	gcaagctaccgtctggaca
	R	cttgcctcagagggtcaatg
IFIT 3	F	gtgctgctacaaggcaaaagt
	R	tcatctctttatttccactagcttca
GAPDH	F	agccacatcgctcagaca
	R	gcccaatacgaccaaatcc

Supplementary Table 4: Primers for cloning

Name	Strand	Enzyme	Sequence (5' to 3')
pIFIT1 -627/+22	F	*Mlu*I	gcgacgcgtctatttaaactcaaattccatgaac
	R	*Bgl*II	ggaagatctaagctgtgggtgtccttgc
pIFIT2 -1366/+163	F	*Mlu*I	cgacgcgttggggaagccaagaacataag
	R	*Bgl*II	gaagatctagtgcaattctcagctgttcg
pCMV IFIT1	F	*Xho*I	ccgctcgaggtatgagtacaaatggtgatgatcatc
	R	*Not*I	ttttccttttgcggccgcctaaggaccttgtctcagag
pCMV IFIT2	F	*Xho*I	ccgctcgaggtatgagtgagaacaataagaattcc
	R	*Not*I	ttttccttttgcggccgctcattccccattccagcttgatg
pMX IFIT1	F	*Bam*HI	cgcggatccatggcatcaatgcagaagctg
	R	*Not*I	ttttccttttgcggccgcctaaggaccttgtctcagag
pMX IFIT2	F	*Bam*HI	cgcggatccatggcatcaatgcagaagctg
	R	*Not*I	ttttccttttgcggccgctcattccccattccagcttgatg

Enzyme sites are underlined.
